# An investigation into the impact of deleting one copy of the *glutaredoxin-2* gene on diet-induced weight gain and the bioenergetics of muscle mitochondria in female mice fed a high fat diet

**DOI:** 10.1080/13510002.2020.1826750

**Published:** 2020-09-29

**Authors:** Robert Gill, Sarah Mallay, Adrian Young, Ryan J. Mailloux

**Affiliations:** a Department of Biochemistry, Faculty of Science, Memorial University of Newfoundland, St. John’s, Canada; b The School of Human Nutrition, Faculty of Agricultural and Environmental Sciences, McGill University, Sainte-Anne-de-Bellevue, Canada

**Keywords:** Mitochondria, sex dimorphisms, glutathionylation, glutaredoxin-2, ROS, bioenergetics, redox buffering‌, obesity

## Abstract

Our group recently documented that male mice containing a deletion for one copy of the *glutaredoxin-2 (Grx2)* gene were completely protected from developing diet-induced obesity (DIO).

**Objectives:** Here, we conducted a similar investigation but with female littermates.

**Results:** In comparison to our recent publication using male mice, exposure of WT and GRX2+/- female mice to a HFD from 3-to-10 weeks of age did not induce any changes in body mass, circulating blood glucose, food intake, hepatic glycogen levels, or abdominal fat pad mass. Examination of the bioenergetics of muscle mitochondria revealed no changes in the rate of superoxide (O_2_^●−^)/hydrogen peroxide (H_2_O_2_) or O_2_ consumption under different states of respiration or alterations in lipid peroxidation adduct levels regardless of mouse strain or diet. Additionally, we measured the bioenergetics of mitochondria isolated from liver tissue and found that partial loss of GRX2 augmented respiration but did not alter ROS production.

**Discussion:** Overall, our findings demonstrate there are sex differences in the protection of female GRX2+/- mice from DIO, fat accretion, intrahepatic lipid accumulation, and the bioenergetics of mitochondria from muscle and liver tissue.

## 1. Introduction

Mitochondria are cellular hot-spots for the regulation of proteins by S-glutathionylation [1, 2]. This is by virtue of the basic matrix environment which promotes thiol ionization for the modification of proteins with GSH, presence of a high concentration of modifiable protein cysteine thiols (60–90 mM), high levels of glutathione, and the presence of glutaredoxin-2 (GRX2), a thiol oxidoreductase that catalyzes the reversible S-glutathionylation of proteins in response to the redox state of the glutathione pool [1, 3]. Mitochondria harbor a number of glutathionylation targets including Krebs cycle enzymes, respiratory complexes, solute anion carriers, antioxidant defense proteins, and factors involved in the induction of apoptosis and fission/fusion [4]. Reversible protein S-glutathionylation has also been recently identified as a negative feedback regulator for ROS production by pyruvate dehydrogenase (PDH), α-ketoglutarate dehydrogenase (KGDH), and complex I and indirectly prevents H_2_O_2_ genesis through the induction of leaks by the uncoupling proteins (UCP) (reviewed in [4, 5]). Furthermore, the reversible modification of proteins by glutathionylation was recently proposed to serve as a global cellular mechanism for the inhibition of ROS production, thereby serving as a keystone in desensitizing H_2_O_2_ signals [6].

The importance of reversible glutathionylation in the regulation of mitochondrial bioenergetics is underscored by the negative consequences associated with dysfunctional S-glutathionylation reactions. Prolonged inhibition of mitochondrial proteins by glutathionylation in heart tissue results in development of left ventricular cardiomyopathy, fibrosis, and metabolic inflexibility which is associated with a ∼50% decrease mitochondrial oxidative phosphorylation and deactivation of complex I [7]. Prolonged inhibition of complex I by glutathionylation in lens epithelia results in development of cataracts in mice and dysfunctional glutathionylation reactions in the matrix compromises embryonic development [8, 9]. Additionally, small nuclear polymorphisms that lead to a loss-of-function in GRX2 activity correlates with the development of heart disease in humans [10]. Overall, this demonstrates that reversible glutathionylation reactions are integral for the regulation of mitochondrial bioenergetics.

Surprisingly, the partial or complete deletion of the *Grx2* gene in some other tissues can have a protective effect in rodents. Male C57BL6N mice homozygous for the *Grx2* gene display a significant decrease in weight gain when fed a standard chow diet [11]. This was associated with a decrease in gonadal fat pad mass, augmented whole body energy expenditure, and increased respiration due to deglutathionylation and activation of proton leaks throughUCP3 in muscles [11]. Our group recently followed up on these seminal findings and revealed that male mice heterozygous for the *Grx2* gene (*Grx2+/−*) are completely resistant to the development of DIO following exposure to a HFD for seven weeks [5]. This was associated with protection from abdominal fat pad accretion, intrahepatic lipid accumulation and glycogen granule depletion, and maintenance of circulating triglyceride and insulin levels that were comparable to littermates fed a control-matched diet [5]. These effects correlated strongly with a ∼50% decrease in the overall S-glutathionylation of skeletal muscle mitochondrial proteins, which included UCP3 and adenine nucleotide translocase (ANT) [5]. This resulted in the activation of proton leaks through both proteins and an increase in mitochondrial fuel combustion by ∼four-fold [5].

Sex dimorphisms in cellular metabolism and ROS production are prevalent in mammals and humans [12]. These differences are underscored by the pathogenesis of metabolic disorders and aging. Indeed, female rodents and women are more protected from development of metabolic disorders, which has been attributed to the positive effects of 17β-estradiol signaling and its ability to bolster cell redox buffering capacity, limit ROS production, induce mitochondrial proliferation, and augment fat metabolism [12]. As noted above, cell redox signals such as glutathionylation are vital for controlling cell metabolism and mitochondrial bioenergetics. However, only one study has addressed the impact of sex on mitochondrial glutathionylation pathways [13]. Given our observations that male mice containing a partial deletion for the *Grx2* gene are protected from DIO, in the present study, we followed up on our findings described above and investigated the response of female GRX2+/− mice and WT littermates towards high fat feeding for 7 weeks.

## 2. Materials and methods

*Chemicals*: The triglyceride assay kit was obtained from Cederlane. Amplex UltraRed (AUR) reagent and 100 bp protein ladder were obtained from Invitrogen. CPI-613 was purchased from Santa Cruz. ADP, oligomycin, antimycin A, bovine serum albumin (BSA), malonic acid, EGTA, glutathione assay kit, HEPES, horseradish peroxidase (HRP), mannitol, MgCl_2_, NaCl, NADH, oligomycin, palmitoyl-carnitine, ponceau, pyruvic acid, the red Extract-N-Amp PCR kit, malic acid superoxide dismutase (SOD), subtilisin A, succinic acid, and sucrose were all obtained from Sigma.

*Animals and diet*: Animals were cared for in accordance with the principles and guidelines of the Canadian Council on Animal Care and the Institute of Laboratory Animal Resources (National Research Council). All procedures using mice were approved by the Animal Care and Use Committee at Memorial University of Newfoundland and the Facilities Animal Care Committee at McGill University. Male and female mice heterozygous for GRX2 (GRX2+/−) were paired for breeding and housed on a 12-h day/night light cycle and were fed ad libitum with a standard chow diet (Teklad Global 18% Protein Rodent Diet, 2018). Litters were weaned at 3 weeks of age and ear notched. Female mice were genotyped and GRX2+/− mice and wild-type (WT) littermates were either fed a high-fat diet (HFD; Teklad diet TD.06415; fat [lard and soybean oil]: % kcal = 44.8, carbohydrates: % kcal = 36.2, protein: % kcal = 19) or matched control diet (CD, Teklad TD.06416; fat [lard and soybean oil]: % kcal = 10.2, carbohydrates: % kcal = 69.8, protein: % kcal = 20. Composition of the two diets in terms of vitamins and minerals was identical) ad libitum for up to 10 weeks. Mouse mass and food consumption were measured weekly. Blood glucose levels were also measured weekly using a glucometer (blood was collected by leg vein puncture). At 10 weeks of age, mice were euthanized under heavy anesthesia (5% isoflurane) by cardiac puncture. Blood collected from cardiac puncture was immediately placed in an microfuge on ice. Abdominal fat (posterior subcutaneous depots), liver, and muscle (pooled from fore and hindlimb and pectoral muscles) were collected, dried by gently blotting with Kim wipes, and weighed. One lobe of the liver was sectioned and used for histological staining. Pooled muscle tissues and liver were also used for the isolation of mitochondria. Liver sections were subjected to Oil Red-O or Periodic acid Schiff (PAS) staining for the visual detection of intrahepatic neutral lipids and glycogen granules, respectively. Histological staining was conducted by the Medical Education and Laboratory Support Services at the Memorial University of Newfoundland Faculty of Medicine.

*Genotyping:* Genotyping was carried out using the Extract-N-Amp Tissue PCR kit according to the manufacturer's instructions as described in [5]. Primer sequences for the *Grx2* gene were produced by Integrated DNA technologies [Forward: 5’-GAC CTA GCC TAC CAG ACT TGG CTG AAA TTT ATT C-3’ (located in intron 2) Reverse: 5’-CAT AGA CAC TCT TCA CTT TCA AGC CCA CCC TC-3’ and Neo: 5’-CCT ACA TTT TGA ATG GAA GGA TTG GAG CTA CGG G-3’] [14]. PCR samples were electrophoresed in a 1.5% (w/v) agarose gel supplemented with 5 µL SYBR safe and fragment size estimation was performed using the Trackit 100 bp DNA ladder as described in [14]. The amplified *Grx2* sequence in WT mice has a fragment size of 729 bp while the truncated form in the homozygotes is ∼510 bp. Mice heterozygous for the *Grx2* gene contain both fragments [5, 14].

*Blood serum analysis*: Blood collected by cardiac puncture was then placed in EDTA-treated tubes and serum collected by centrifugation at 2000×*g* for 10 min at 4°C. Serum was then stored at −80°C. Triglycerides (Wako; CA10752-444) and total glutathione, GSH, and GSSG (Sigma; CS0260-1KT) levels were measured according to the manufacturer's instructions.

*Isolation of mitochondria*: All steps were performed on ice or at 4°C. Pooled muscles and livers were placed either in basic medium (BM; KCl; 140 mM, HEPES; 20 mM, EGTA; 1 mM, and MgCl_2_; 5 mM, pH 7.0 with 6 N HCl) or MESH buffer (220 mM mannitol, 1 mM EGTA, 70 mM sucrose, 10 mM HEPES, pH 7.2 with 6 N HCl), respectively, and then rinsed several times to remove excess blood. Mitochondria were then isolated as described in [5, 15]. Protein equivalents to mitochondria were estimated with the Bradford assay using BSA as the standard. Isolated mitochondria were then stored on ice at equivalents of ∼10 mg/mL for muscle and ∼16 mg/mL for liver, respectively, and used immediately for ROS kinetics and respiration assays. Remaining mitochondria were then stored at −80 degrees for 4-hydroxy-2-nonenal adducts adduct level analysis.

*4-hydroxy-2-nonenal protein adducts assay*: Muscle and liver mitochondria were diluted to 0.5 mg/mL in either BM or MESH and then 4-hydroxy-2-nonenal protein adducts were estimated according to the manufacturer's instructions (Abcam).

*Estimation of O_2_^●−/^H_2_O_2_ production*: Skeletal muscle and liver mitochondria were diluted to 1.5 mg/mL or 5 mg/mL, respectively, in ice cold BM or MESH and stored on ice prior to commencing assays. Samples were then diluted to 0.15 mg/mL (muscle) and 0.5 mg/mL (liver) in the individual wells of black clear-bottom 96-well plate containing room temperature BM and then mitochondria were allowed to equilibrate for a few minutes. Wells were then supplemented with HRP (3 U/mL), SOD1 (25 U/mL), and AUR (10 nM). Reactions were then initiated by adding substrate; pyruvate (50 µM) and malate (50 µM), succinate (50 µM), or palmitoyl-carnitine (20 µM). Note that for experiments using palmitoyl-carnitine, mitochondria were pre-incubated for 5 min at 25°C in carnitine (2 mM) prior to adding AUR reagents. A cocktail of ROS generation inhibitors was utilized to ensure the fluorescent signal corresponded to O_2_^●−/^H_2_O_2_ production. Inhibitors were selected based on recent findings that have demonstrated the site for ROS production by mitochondria can vary significantly depending on which fuel is being combusted [5, 16]. Mitochondria were pre-treated with the various inhibitors for 10 min at 25°C prior to starting assays. Note that for experiments with palmitoyl-carnitine, mitochondria were pre-loaded with carnitine prior to inhibitor treatment. Changes in resorufin fluorescence were followed at excitation/emission wavelengths 565 nm/600 nm for 5 min with reads taken once every 30 s using a SpectraMax M5 plate reader. Values were normalized to background fluorescence and protein content.

*Mitochondrial bioenergetics*: Skeletal muscle and liver mitochondria were diluted to 0.2 mg/mL or 0.5 mg/mL, respectively, in an Oxytherm (Hansatech) chamber containing respiration buffer (BM + 0.5% w/v BSA, KH_2_PO_4_; 10 mM, and MgCl_2_; 2 mM, pH 7.4 for muscle or MESH + 0.5% w/v BSA, KH_2_PO_4_; 10 mM, and MgCl_2_; 2 mM, pH 7.4 for liver). Once a baseline oxygen consumption was established, pyruvate (10 mM) and malate (2 mM) or succinate (5 mM) were injected into the chamber to initiate state 2 respiration. After a few minutes, state 3 respiration was induced by injecting ADP (1 mM) into the reaction chamber. Oligomycin (4 µg/mL) was then added once ADP was exhausted to inhibit contaminating ATPases and complex V for the measurement of state 4 respiration (proton leak-dependent respiration). Finally, the reaction was ended with the addition of antimycin A (4 µM).

*Data analysis*: Graph pad prism 6 software was utilized for all statistical analysis. All data is represented as the mean ± standard error of mean (SEM) with N ≥ 4. One-way and two-way analysis of variance (ANOVA) with a Tukey's post-hoc test was employed for all experimental results. Statistical significance was represented as follows: * or #; *P* ≤ 0.05, ** or ##; *P* ≤ 0.01, *** or ###; *P* ≤ 0.001, **** or ####; *P* ≤ 0.0001.

## 3. Results and discussion

### 3.1 WT female mice are resistant to DIO and deleting one of the *Grx2* genes has no protective effects

**Figure 1.**
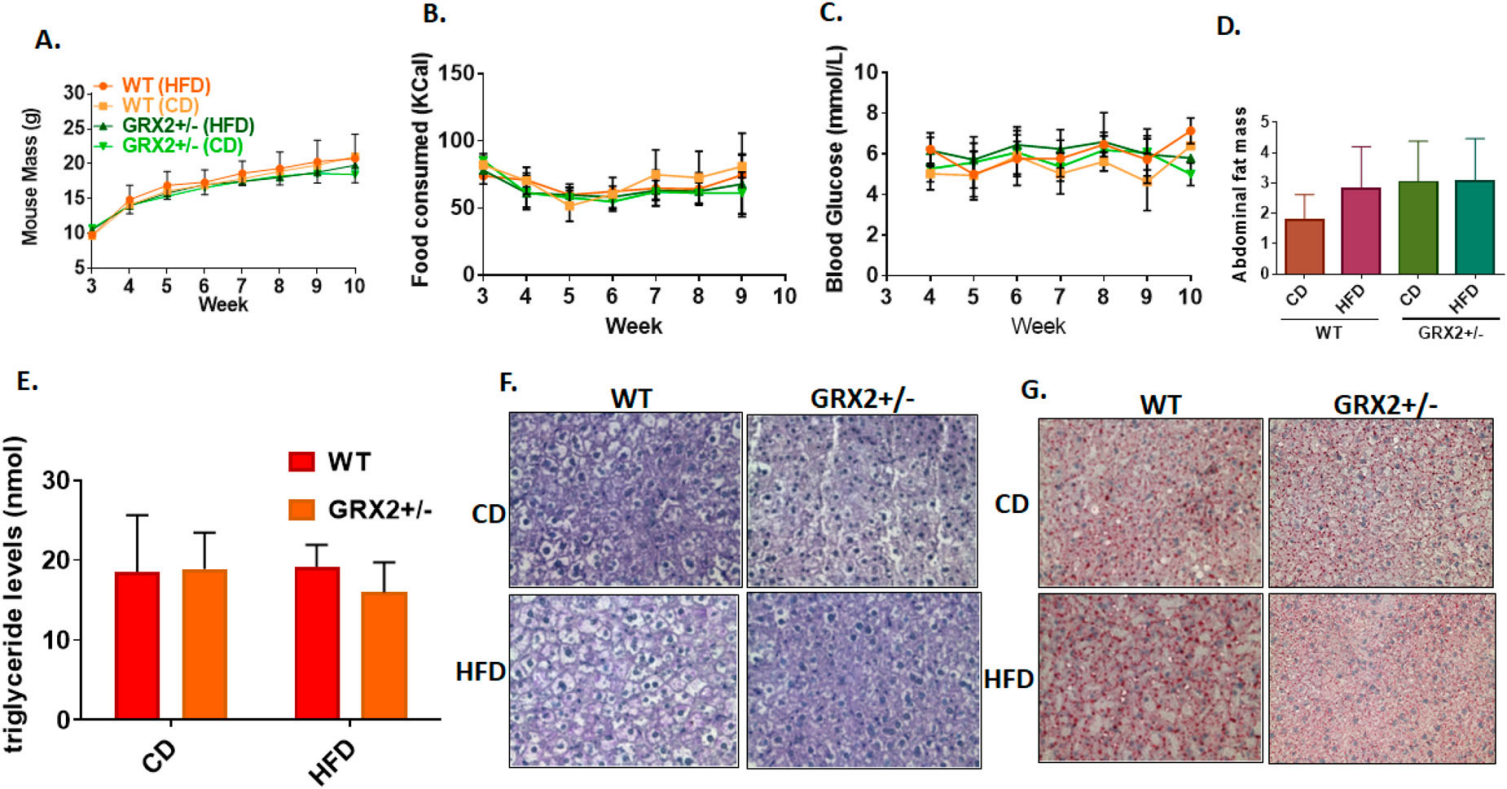
Female mice are resistant to DIO and deletion of the *Grx2* gene is not required for protection from HFD-induced weight gain. (A) Mass of mice fed a CD and HFD (*N* = 8). (B) Food intake (*N* = 8). (C) Blood glucose concentration (*N* = 4–6). (D) Abdominal fat pad mass (*N* = 4). Mean ± SEM, two-way ANOVA with a Tukey's post-hoc test for A–C and one-way ANOVA with a Tukey's post-hoc test for D. (E) Serum triglyceride levels (*N* = 4), mean ± SEM, one-way ANOVA with a Tukey's post-hoc test. (F) Liver glycogen granule content (*N* = 3). (G) Intrahepatic staining of neutral lipids with Oil Red-O (*N* = 3).

As noted above, we recently reported that male mice heterozygous for the *Grx2* gene were completely protected from DIO when fed a HFD from three to ten weeks of age due to an augmentation of fat combustion and proton leaks in muscle [5]. Our group also recently observed that there is a sex dimorphism in the regulation of mitochondrial bioenergetics by GRX2-mediated signaling [13]. Based on these findings we decided to examine if there was a sex dimorphism associated with the GRX2-mediated protection of mice from DIO. Female mice were fed a CD and HFD immediately following genotyping and sex identification from 3 to 10 weeks of age. We observed no significant changes in weight gain in WT mice fed a HFD when compared to WT and GRX2+/− littermates exposed to the control-matched diet (Figure 1A). Additionally, deleting one of the *Grx2* genes did not induce any significant changes in weight gain over the seven-week period (Figure 1A). No significant differences in food consumption were observed indicating that differences in weight gain was not attributed to alterations in food intake (Figure 1B) and there were no significant genotype or diet effects on blood glucose levels (Figure 1C). Finally, there was no diet or genotype effect on fat pad mass (Figure 1D).

Next, we measured circulating triglyceride levels and conducted histological analyses of intrahepatic neutral lipids and glycogen granule levels. Measurements conducted on WT female mice fed a HFD revealed that serum triglycerides that were almost identical to that of littermates fed a control diet (Figure 1E). Furthermore, serum from GRX2+/− mice fed either diet also contained triglyceride levels that were similar to WT littermates fed either the CD or HFD (Figure 1E). Histological analyses revealed no visible differences in intrahepatic glycogen granule content (Figure 1F). However, we did note that feeding WT female mice a HFD resulted in the accumulation of intrahepatic lipids when compared to GRX2+/− littermates fed either CD or HFD (Figure 1G). This would suggest that a 50% decrease in GRX2 protein levels can potentially protect female mice from the development of fatty liver disease. The results presented above are not surprising given that several studies conducted on human and rodent models have demonstrated that women and female rodents are more resistant to the development of metabolic disorders (reviewed in [12, 17, 18]). This is underscored by observations showing that menopause or short-term ovariectomy abolishes protection from the development of metabolic syndrome [19, 20]. However, what is unique here is that unlike male mice, female mice are not as reliant on the GRX2-glutathionylation axis for protection from DIO and the development of related disorders. To our knowledge, only two studies have demonstrated to date that there is a sex dimorphism in cellular S-glutathionylation [13, 21]. Here, we have found that deleting one of the two *Grx2* genes is not required to protect female mice from DIO but may prevent intrahepatic lipid accretion following prolonged high fat feeding. Therefore, female rodents have a higher capacity for protection from DIO in comparison to male littermates but do not contain the capacity to prevent intrahepatic lipid accumulation.

### 3.2 Impact of high fat feeding on oxidative stress markers

**Figure 2.**
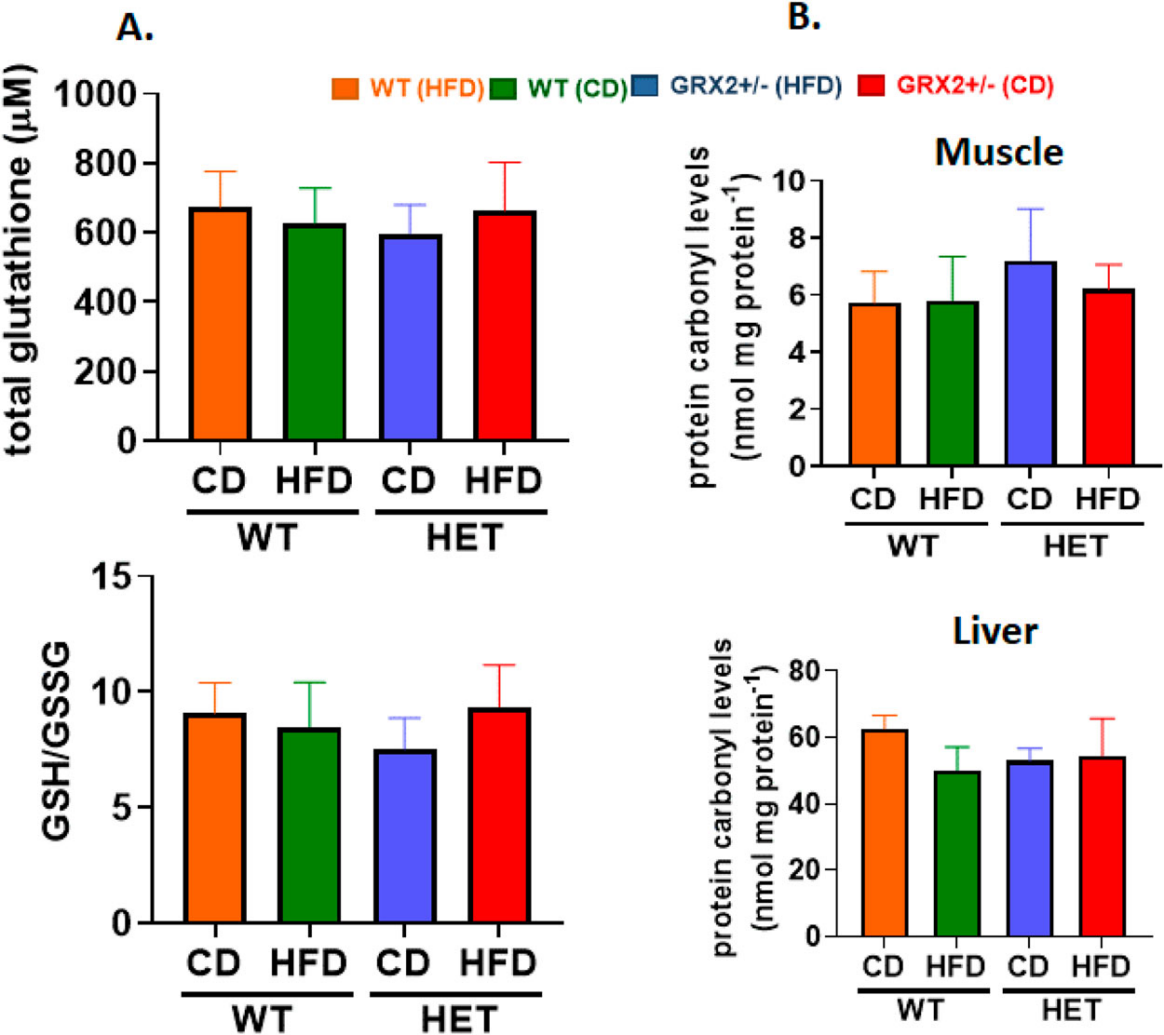
WT and GRX2+/− female mice fed a CD or HFD do not display any changes in glutathione redox buffering capacity or development of oxidative stress. (A) Total serum glutathione levels and the ratio of GSH to GSSG. (B) Mitochondria from muscle and liver tissue were isolated and examined for the presence of 4-hydroxy-2-nonenal/protein adducts. *N* = 4, mean ± SEM, one-way ANOVA with a Tukey's post-hoc test.

We had previously shown that male mice heterozygous for the *Grx2* gene were protected from HFD-induced oxidative stress and damage by increasing redox buffering capacities in muscle and in circulation [5]. Additionally, WT male mice fed a HFD displayed a significant increase in 4-HNE adducts, an effect that was abolished when one of the two *Grx2* genes was deleted [5]. Intriguingly, we observed no differences in the GSH/GSSG ratio or the availability of circulating total glutathione in WT and GRX2+/− female mice fed either diet (Figure 2A). No differences in the amount of 4-HNE in muscle or liver mitochondria collected from WT and GRX2+/− female mice fed a CD or HFD was also observed (Figure 2B). This sex dimorphism can be attributed to observations demonstrating that mitochondria isolated from various tissues from female rodents have better ROS handling capabilities due to more efficient electron flow through the respiratory chain, increased proton leaks, and higher antioxidant defenses (reviewed in [12]). These sex differences in antioxidant availability are further underscored in the present study and our previously published findings where it was found that the circulating total glutathione pool was ∼four-fold higher in female mice fed either diet (Figure 2A) when compared to WT and GRX2+/− male mice [5]. Additionally, the circulating glutathione pool was ∼four-fold more reduced in female mice fed either diet (Figure 2A) in contrast to WT male mice [5]. It was posited in a recent review that protein S-gluthationylation is required to serve as a global inhibitor for ROS production to preserve and restore cell redox buffering capacity following oxidation of antioxidant defenses [6]. This is likely the case in mitochondria from male mice where glutathionylation following glutathione pool oxidation inhibits ROS production by PDH, KGDH, and complex I in liver, heart and muscle mitochondria, respectively (reviewed in [6]). Furthermore, it has been documented that GRX2 is required to deglutathionylate these proteins [2, 14]. However, this does not appear to be the case for female mice, which may be attributed to the superior redox buffering capacity of mitochondria (e.g. the higher levels of reduced glutathione provides an enhanced redox buffering capacity which may negate the need for using glutathionylation as a negative feedback loop to inhibit ROS production). The robustness of the redox buffering capacity of mitochondria from female mice may be attributed to 17β-estradiol signaling, which results in the activation of genes involved in antioxidant defenses and mitochondrial proliferation [12, 22]. A recent study also showed that 17β-estradiol intercalates within the mitochondrial inner membrane improving respiration and ROS handling [19]. Our group also showed that GRX2 and reversible protein S-glutathionylation was not required to control ROS production by liver and muscle mitochondria isolated from female mice due to higher proton leaks and better ROS handling [13]. This is in contrast to several other studies showing muscle, cardiac, and liver mitochondria are highly dependent on reversible S-glutathionylation serving as a negative feedback loop for the regulation of ROS genesis [13, 14, 23]. Thus it is likely that female mice are not as reliant on using glutathionylation to inhibit mitochondrial ROS production due to the higher availability of antioxidant defenses, superior redox buffering capacities and better ROS handling abilities when compared to male mice.

### 3.3 Deleting one of the *Grx2* genes or feeding mice a HFD does not alter the bioenergetics of muscle mitochondria

**Figure 3.**
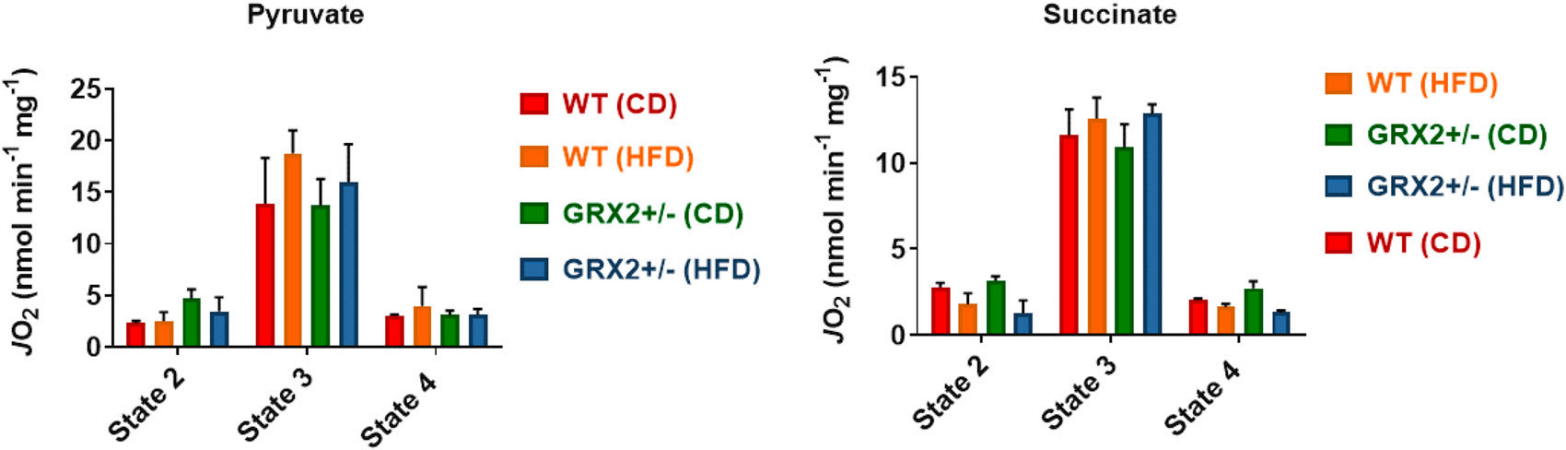
Muscle mitochondria from WT and GRX2+/− female mice fed a CD or HFD do not display any significant changes in bioenergetics. Skeletal muscle mitochondria were isolated and the different states of respiration were measured using an Oxytherm Clark-type electrode. State 2 was initiated by the addition of substrate followed by the induction of state 3 respiration through the treatment of mitochondria with ADP. State 4 respiration was induced once mitochondria had exhausted their ADP supply and were treated with oligomycin. *N* = 4, mean ± SEM, one-way ANOVA with a Tukey's post-hoc test.

We had previously reported that the rate of state 3 and proton leaks-dependent respiration increased by up to ∼four-fold in muscle mitochondria from GRX2+/− male mice energized with pyruvate, succinate, or palmitoyl-carnitine [5]. Muscle mitochondria isolated from WT or GRX2+/− female rodents fed a CD or HFD displayed no significant changes in state 2 (substrate only), ADP-stimulated respiration (state 3), or proton leaks-dependent respiration (state 4) when energized with pyruvate (Figure 3). Similar observations were made when mitochondria were metabolizing succinate alone (Figure 3). These findings are in line with a previous study published by our group where we showed that there were sex dependent differences in the regulation of mitochondrial bioenergetics by the GRX2-glutathionylation signaling axis [13]. For instance, it was found that partial or full deletion of the *Grx2* genes had no impact on ADP-stimulated or proton leaks-dependent respiration in muscle mitochondria from female mice whereas there was an inverse relationship between the deletion of the gene and the rate of respiration in male samples (e.g. there was a dose-dependent relationship between GRX2 availability and the rate of respiration) [13]. Additionally, rates of state 3 respiration were ∼two-fold higher in WT and *Grx2* heterozygous and homozygous muscle mitochondria from female littermates when compared to samples from WT males [13]. However, the deletion of one or both of the *Grx2* genes in male mice led to a significant increase in state 3 respiration culminating with a rate of O_2_ consumption in the full GRX2 knockout muscle mitochondria that matched that of the female mice [13]. This increase in respiration is associated with the deglutathionylation and activation of several mitochondrial proteins involved in proton leaks and fuel metabolism, including UCP3 and ANT [5]. By contrast, we did not observe any significant changes in proton leak-dependent respiration in WT and GRX2+/− female mice fed either the CD or HFD, regardless of which substrate is being metabolized (Figure 3). In our previous study with male mice, it was found that the deglutathionylation of UCP3 and ANT was required to augment fuel metabolism in muscles for protection from DIO [5]. However, as demonstrated here and in a second previously published study [13], fuel metabolism in the muscle mitochondria from female mice is several fold higher when compared to males, which has also been reported for both rodents and humans in previous studies [12, 24]. Additionally, it had been previously recorded that muscle mitochondria from female rodents and humans also display higher rates of proton leaks which would (1) indicate that UCP3 and ANT are potentially more active and (2) account for the higher rates of fuel metabolism in female muscles [12, 24]. Therefore, although not measured here, it is likely that UCP3 and ANT are more deglutathionylated and thus more active, which would partially account for the higher rates of fuel metabolism in the muscles of female rodents.

**Figure 4.**
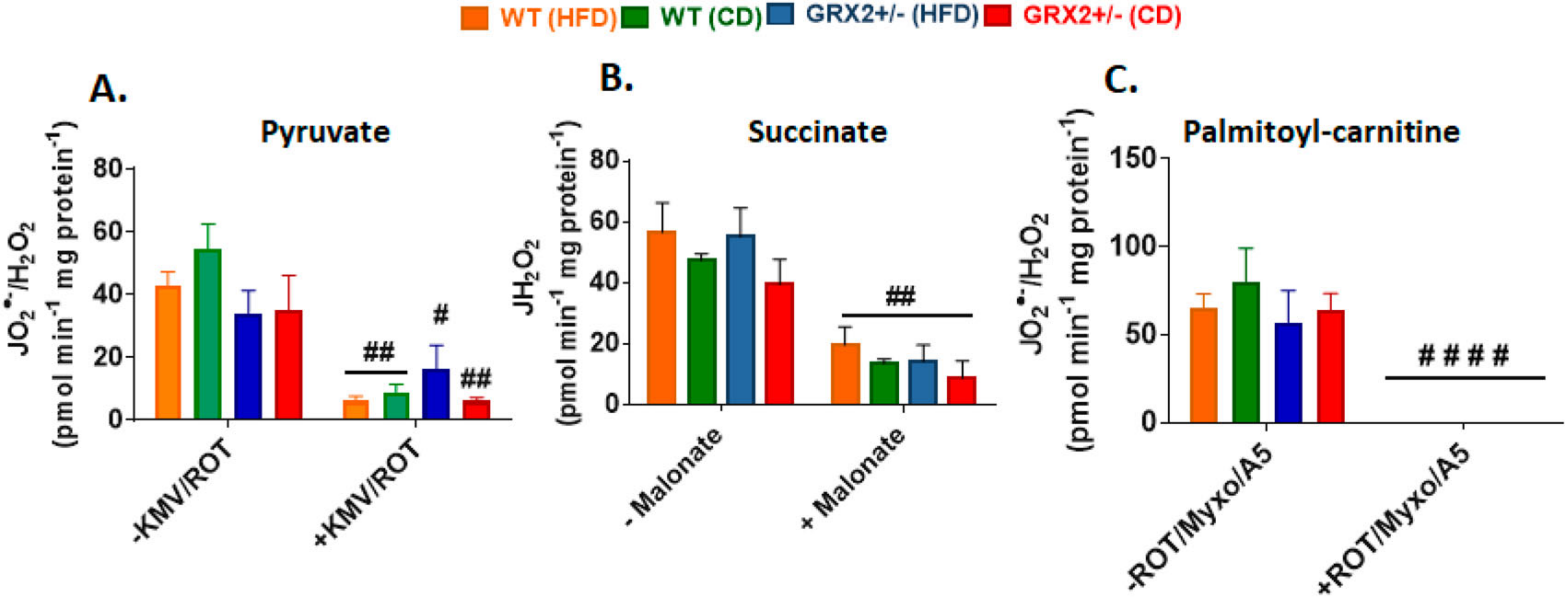
Muscle mitochondria from female mice do not display significant differences in the rate of H_2_O_2_ production. Skeletal muscle mitochondria were isolated, energized with pyruvate/malate, succinate, or palmitoyl-carnitine, and then the rate of H_2_O_2_ production was measured over the course of 5 min using Amplex UltraRed. Mitochondria were treated with various inhibitors for ROS production to ensure changes in the fluorescent signal were associated with H_2_O_2_ generation. *N* = 4, mean ± SEM, one-way ANOVA with a Tukey's post-hoc test.

We found no significant differences in O_2_^●−^/H_2_O_2_ production between genotypes and diet, regardless of whether mitochondria were energized with pyruvate, succinate, or palmitoyl-carnitine (Figure 4). Furthermore, pre-incubation of mitochondria with various inhibitors significantly decreased or completely abolished O_2_^●−^/H_2_O_2_ production indicating changes in the fluorescent signal were due to the oxidation of Amplex UltraRed to resorufin by hydrogen peroxide (Figure 4). This is in contrast to our previous studies that demonstrated mitochondria collected from the muscles of male GRX2+/− mice display significantly higher rates of ROS production [5, 13]. Intriguingly, muscle mitochondria from female mice yielded rates of O_2_^●−^/H_2_O_2_ production that were several fold higher than their male counterparts, regardless of the substrate being metabolized [13]. Deleting one of the *Grx2* genes in male mice increased ROS production but the rates were still lower in comparison to muscle mitochondria isolated from female littermates. Although this may seem like a detrimental effect, it is far more likely that the increased ROS production by these mitochondria is a benefit since it would trigger adaptive signaling responses that bolster antioxidant defenses and redox buffering capacity. This is underscored by observations demonstrating that several tissues, including muscles, in female rodents and humans have superior redox buffering capacity and ROS handling and protection from oxidative distress [12, 19]. Therefore, it is likely that muscle from male mice are more reliant on reversible protein S-glutathionylation to control mitochondrial ROS production due to a lower redox buffering capacity when compared to female mice. There may also be compensatory changes in the availability of the cytosolic isoform of GRX2, GRX1, in female muscles as well; however, we did previously show that deleting the GRX2 gene does not alter GRX1 expression in male mice [14]. Another possibility is a potential compensatory increase mitochondria-targeted glutathione S-transferases (GST), which plays a part in driving glutathionylation reactions as well. These factors may contribute to the lack of effect observed in female mice heterozygous for the *Grx2* gene and thus deserves further investigation.

### 3.4. Liver mitochondria from female mice heterozygous for Grx2 display improved fuel combustion and respiration

**Figure 5.**
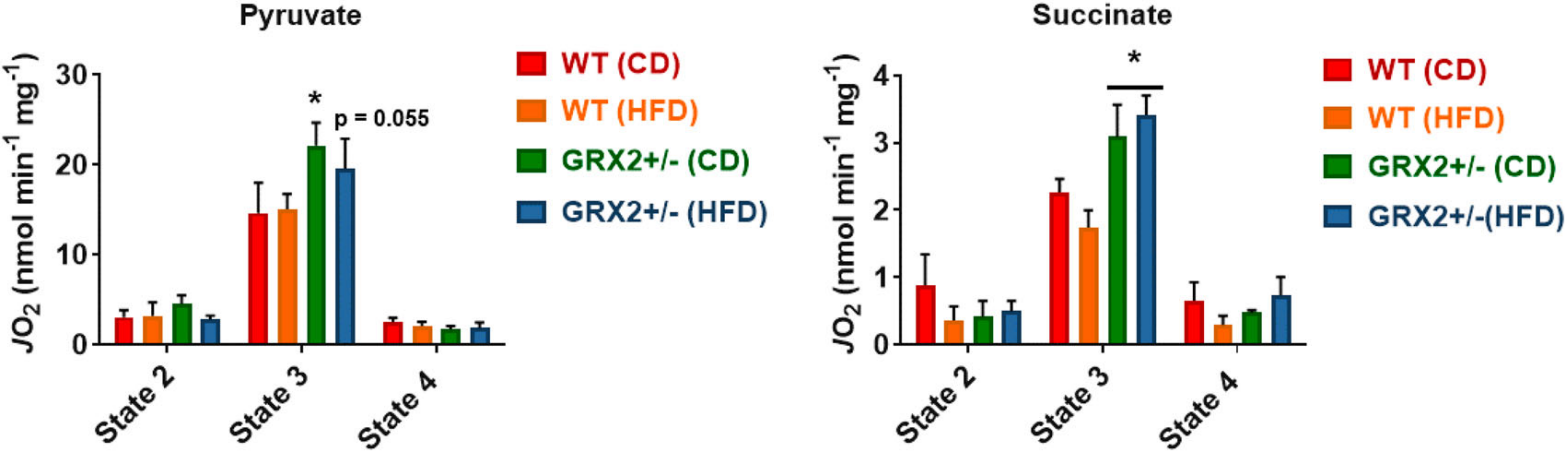
Liver mitochondria from GRX2+/− female mice fed a CD or HFD have significantly higher rates of ADP-stimulated respiration. Liver mitochondria were isolated and the different states of respiration were measured using an Oxytherm Clark-type electrode. State 2 was initiated by the addition of substrate followed by the induction of state 3 respiration through the treatment of mitochondria with ADP. State 4 respiration was induced once mitochondria had exhausted their ADP supply and were treated with oligomycin. *N* = 3–5, mean ± SEM, one-way ANOVA with a Tukey's post-hoc test.

**Figure 6.**
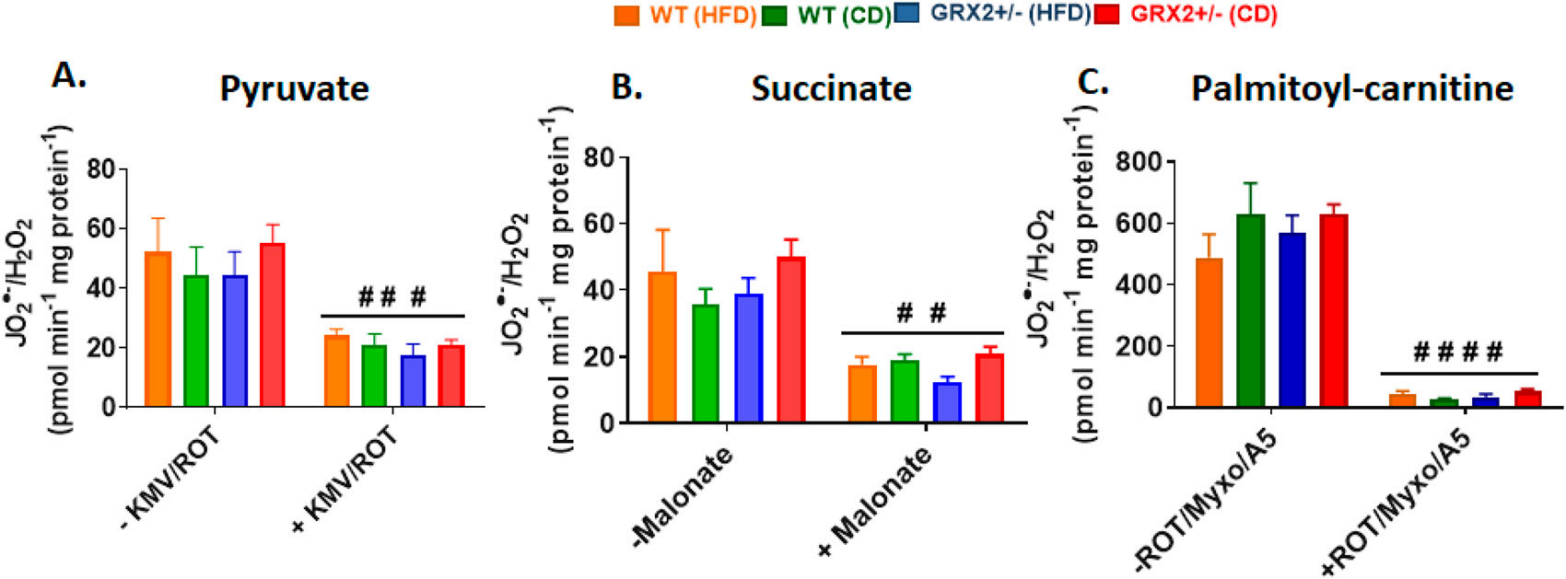
Liver mitochondria from female mice do not display significant differences in the rate of H_2_O_2_ production. Mitochondria were isolated, energized with pyruvate/malate, succinate, or palmitoyl-carnitine, and then the rate of H_2_O_2_ production was measured over the course of 5 min using Amplex UltraRed. Mitochondria were treated with various inhibitors for ROS production to ensure changes in the fluorescent signal were associated with H_2_O_2_ generation. *N* = 4, mean ± SEM, 1-way ANOVA with a Tukey's post-hoc test.

In Figure 1(G), we observed that feeding female WT littermates a HFD resulted in lipid droplet accretion. By contrast, mice heterozygous for the *Grx2* gene displayed intrahepatic neutral lipid levels that were like female littermates fed a control-matched diet. Our group recently observed that liver mitochondria collected from female mice heterozygous and homozygous for GRX2 displayed augmented ADP-stimulated and proton leaks [13]. By contrast, this effect was not observed in male littermates [13]. Intriguingly, deleting the *Grx2* gene elevated state 3 respiration in liver mitochondria from female mice by ∼threefold and ∼twofold, respectively, in comparison to WT females and male littermates [13]. Based on this, we hypothesized that female mice heterozygous for *Grx2* were protected from diet-induced intrahepatic lipid accumulation through an increase in mitochondrial fuel combustion in liver tissue. Deleting one of the two *Grx2* genes augmented state 3 respiration in liver mitochondria isolated from female mice fed the control-matched diet (Figure 5). Additionally, there was a trend for increased state 3 respiration in liver mitochondria isolated from mice fed a HFD but this was not significant when compared to the WT control diet group (Figure 5). Similar observations were made when succinate served as the substrate, revealing a significant increase in O_2_ consumption in female GRX2+/− mitochondria (Figure 5). However, despite the increase in respiration, we observed no differences in ROS production by liver mitochondria oxidizing pyruvate, succinate, or palmitoyl-carnitine, which may be attributed to the higher redox buffering capacity of mitochondria from the livers of female mice (Figure 6). Recent work has demonstrated that female rodents fed a high fat diet for a short period of time develop fatty liver disease due to dysfunctional mitochondrial bioenergetics in hepatic tissue [25]. This was associated with the rapid intrahepatic accumulation of lipids and the induction of oxidative stress [25]. Although we did not observe any differences in ROS production or the induction of oxidative distress in hepatic mitochondria, we did find that deleting the *Grx2* gene induced a small but statistically significant increase in mitochondrial fuel metabolism and respiration which likely provided some protection from intrahepatic fat accretion. Taken together, our findings demonstrate that reducing the availability of GRX2 by ∼50% in livers can augment fuel combustion and provide some protection from hepatic lipid accumulation.

Sex dimorphisms are prevalent traits in organisms that result in important biochemical, epigenetic, and genetic differences in males and females. The manifestation of these traits has an impact on longevity and the development of various disorders. It is documented that women and female rodents display enhanced protection from the development of these disorders due to the signaling properties and chemistry of 17β-estradiol (reviewed in [12, 24]). Additionally, some of this increased protection is afforded through the induction of adaptive signaling pathways that promote improved redox buffering capacity, protection from oxidative stress, and more efficient mitochondrial fuel combustion and respiration (reviewed in [12]).

Reversible protein S-glutathionylation is considered to be a vital mechanism for the negative regulation of H_2_O_2_ second messaging through the inhibition of cellular metabolism [6]. Recent work by our group and others has demonstrated that manipulating these pathways can protect male rodents from the development of DIO and other disorders such as fatty liver disease [5, 26, 27]. However, to our knowledge, only two studies have taken into account the impact of sex on glutathione signaling [13, 21]. Here, we have expanded on our previous publications by demonstrating that there is a sex dimorphism associated with GRX2-glutathionylation signaling in muscle and liver mitochondria. Indeed, we show that loss of GRX2 does not have any effect on mitochondrial respiration and ROS production in muscles and nor is it required to protect female mice from DIO and fat pad accretion. This is in contrast to male mice where deleting the *Grx2* gene protected mice from DIO and fatty liver disease through the augmentation of muscle fuel metabolism [5]. We attribute this sex dimorphism to more efficient mitochondrial respiration and fuel combustion and a more robust redox buffering capacity in female mice which limits oxidative stress and cellular damage, negating the need to rely on glutathionylation pathways for the inhibition of mitochondrial ROS production. Another important and unique observation made in this study was that WT female mice accumulate intrahepatic fat droplets following high fat feeding for several weeks. Intriguingly, this was prevented in mice heterozygous for the *Grx2* gene, an effect that was associated with a significant increase in hepatic mitochondrial respiration and fuel combustion. This points to the possibility that promoting the deglutathionylation of mitochondrial proteins in female liver mitochondria may serve as a novel means for treating/preventing fatty liver disease, a line of questioning that our group will begin investigating.
